# An individualized decision between physical therapy or surgery for patients with degenerative meniscal tears cannot be based on continuous treatment selection markers: a marker-by-treatment analysis of the ESCAPE study

**DOI:** 10.1007/s00167-021-06851-x

**Published:** 2022-02-05

**Authors:** Julia C. A. Noorduyn, Victor A. van de Graaf, Nienke W. Willigenburg, Gwendolyne G. M. Scholten-Peeters, Ben W. Mol, Martijn W. Heymans, Michel W. Coppieters, Rudolf W. Poolman, for the ESCAPE Research Group, V. A. B. Scholtes, E. L. A. R. Mutsaerts, M. R. Krijnen, D. F. P. van Deurzen, D. J. F. Moojen, C. H. Bloembergen, A. de Gast, T. Snijders, J. J. Halma, D. B. F. Saris, N. Wolterbeek, C. Neeter, G. M. M. J. Kerkhoffs, R. W. Peters, I. C. J. B. van den Brand, S. de Vos-Jakobs, A. B. Spoor, T. Gosens, W. Rezaie, D. J. Hofstee, B. J. Burger, D. Haverkamp, A. M. J. S. Vervest, T. A. van Rheenen, A. E. Wijsbek, E. R. A. van Arkel, B. J. W. Thomassen, S. Sprague, B. W. J. Mol, M. Schavemaker, J. Wolkenfelt, M. Teuwen, I. K. Butter, M. W. van Tulder

**Affiliations:** 1 Department of Orthopedic Surgery, Joint Research, OLVG Amsterdam, Oosterpark 9, 1091 AC Amsterdam, The Netherlands; 2 Department of Human Movement Sciences, Faculty of Behavioural and Movement Sciences, Vrije Universiteit Amsterdam, Amsterdam Movement Sciences, Amsterdam, The Netherlands; 3 Department of Orthopedic Surgery, St. Antonius Ziekenhuis, Utrecht, The Netherlands; 4 Department of Obstetrics and Gynecology, School of Medicine, Monash University, 246 Clayton Road, Clayton, Melbourne, VIC 3168 Australia; 5 Department of Epidemiology and Biostatistics, VU University Medical Centre, Amsterdam, The Netherlands; 6 Menzies Health Institute Queensland, Griffith University, Brisbane and Gold Coast, Australia; 7 Department of Orthopedic surgery, Leiden University Medical Centre, Leiden, The Netherlands

**Keywords:** Knee, Meniscus, Rehabilitation, Orthopedics, Exercise, Prediction, Individualized, Healthcare

## Abstract

**Purpose:**

Marker-by-treatment analyses are promising new methods in internal medicine, but have not yet been implemented in orthopaedics. With this analysis, specific cut-off points may be obtained, that can potentially identify whether meniscal surgery or physical therapy is the superior intervention for an individual patient. This study aimed to introduce a novel approach in orthopaedic research to identify relevant treatment selection markers that affect treatment outcome following meniscal surgery or physical therapy in patients with degenerative meniscal tears.

**Methods:**

Data were analysed from the ESCAPE trial, which assessed the treatment of patients over 45 years old with a degenerative meniscal tear. The treatment outcome of interest was a clinically relevant improvement on the International Knee Documentation Committee Subjective Knee Form at 3, 12, and 24 months follow-up. Logistic regression models were developed to predict the outcome using baseline characteristics (markers), the treatment (meniscal surgery or physical therapy), and a marker-by-treatment interaction term. Interactions with *p* < 0.10 were considered as potential treatment selection markers and used these to develop predictiveness curves which provide thresholds to identify marker-based differences in clinical outcomes between the two treatments.

**Results:**

Potential treatment selection markers included general physical health, pain during activities, knee function, BMI, and age. While some marker-based thresholds could be identified at 3, 12, and 24 months follow-up, none of the baseline characteristics were consistent markers at all three follow-up times.

**Conclusion:**

This novel in-depth analysis did not result in clear clinical subgroups of patients who are substantially more likely to benefit from either surgery or physical therapy. However, this study may serve as an exemplar for other orthopaedic trials to investigate the heterogeneity in treatment effect. It will help clinicians to quantify the additional benefit of one treatment over another at an individual level, based on the patient’s baseline characteristics.

**Level of evidence:**

II.

**Supplementary Information:**

The online version contains supplementary material available at 10.1007/s00167-021-06851-x.

## Introduction

Marker-by-treatment analyses are promising new methods in internal medicine [8], but have not yet been implemented in orthopaedics. Results from randomized clinical trials (RCTs) do not account for the heterogeneity in treatment effect and, therefore, RCT-based treatment recommendations are not always applicable to the individual patient [13, 18, 23]. The more conventional prognostic models identify the association between a prognostic marker and a good or poor treatment response. However, to select the best treatment for an individual patient it is important to quantify the benefit of one treatment over the other [8]. Previous marker-by-treatment analysis provided clinicians an evidence based method to select the best treatment for ovulatory infertile women [30]. In middle aged and older patients with a meniscal tear, the results from RCTs show that meniscal surgery has no clinical advantage over non-surgical treatment (such as physical therapy) or placebo surgery. However, meniscal surgery is associated with higher societal and healthcare costs, and higher risk of serious adverse events [2, 21, 28]. The number of surgeries slowly decreases, but surgery is still regularly performed for degenerative meniscal tears [20]. This is partly explained by the belief among some orthopaedic surgeons and patients that surgery is necessary to regain normal knee function in a subgroup of patients [4]. This view is based on a subgroup of non-responders to conservative treatment in RCTs [1]. [10] Several studies recommend to explore the heterogeneity of treatment outcome to better understand underlying factors which influence individual treatment effects [13, 18, 23]. Previous studies tried to define these subgroups [19, 25]. However, neither multivariable prognostic models [16, 19] nor surgeons’ personal predictions were able to accurately predict treatment outcome [25].

With a marker-by-treatment analysis, the influence of baseline information on the treatment effect can be determined [8, 13]. These predictive factors, or treatment selection markers, represent baseline information regarding patient characteristics, physical and radiological examination findings or patient reported outcomes. The relevant interactions between the baseline characteristics (markers) and treatment outcome can be plotted in a marker-by-treatment predictiveness curve [8]. The analysis provides specific cut-off points that can potentially identify the superior intervention of two interventions. The baseline characteristics that can accurately differentiate between the outcome between interventions are considered relevant treatment selection markers. These treatment selection markers can guide personalized treatment choices, based on a patient’s individual characteristics [8].

Previous prognostic models were unable to accurately predict treatment outcome. Therefore, this study aimed to introduce this novel approach in orthopaedic research and to identify relevant treatment selection markers that affect treatment outcome following meniscal surgery or physical therapy in patients with degenerative meniscal tears. Analysing patient’s baseline characteristics using this method can help clinicians to select the treatment that is potentially the most beneficial for an individual patient.

## Materials and methods

The Medical Research Ethics Committees United (MEC-U; NL44188.100.13) approved the ESCAPE trial and the trial was registered (clinincaltrials.gov: NCT01850719 and The Netherlands Trial Register: NTR3908). All patients provided written informed consent before randomization.

To identify potential treatment selection markers, the data from the ESCAPE trial were used. The ESCAPE trial is a multi-center RCT comparing meniscal surgery with physical therapy in patients over 45 years old with a symptomatic degenerative meniscal tear who do not experience locking of the knee [26]. Patients were randomly allocated to meniscal surgery or physical therapy. Exclusion criteria were severe osteoarthritis (Kellgren and Lawrence score of 4, presenting significant osteophytes, joint-space narrowing, sclerosis, and bone ends abnormality) [11], a body mass index (BMI) > 35 kg/m^2^, prior surgery to the index knee (with the exception of diagnostic arthroscopic surgery), or clinically relevant anterior or posterior cruciate ligament insufficiency. Meniscal surgery, in which the damaged part of the meniscus was removed was performed within 6 weeks after randomization. Physical therapy which consisted of a predefined incremental exercise protocol, consisting of 16 sessions during eight weeks (Supplement 1) [27]. For patients with persistent knee symptoms after the intervention, additional physical therapy sessions could be attended or a delayed meniscectomy could be planned, depending on a shared decision after consultation with the orthopaedic surgeon. Further details of the interventions can be found in the study protocol of the ESCAPE trial [26, 27].

### Selection of baseline characteristics for treatment selection

Baseline characteristics were preselected as possible treatment selection markers from an extensive list of baseline variables that were available (Supplement 2). First, a literature search was conducted to identify factors associated with the treatment outcome in patients with a meniscal tear. The search strategy can be found in Supplement 3. Second, an electronic survey was sent to an expert panel of orthopaedic surgeons (*N* = 24), physical therapists (*N* = 22) and patients (*N* = 10) who were involved in the ESCAPE trial to identify the most relevant treatment selection factors according to their opinion. The final selection of baseline characteristic that were analysed as potential treatment selection markers consisted of variables with a continuous outcome that were identified by the literature and/or chosen by the expert panel as variables associated with the treatment outcome [12].

Potential treatment selection markers included patients’ demographics (age, education level, BMI), patient reported outcome measures (the International Knee Documentation Committee Subjective Knee Form (IKDC) for knee function, pain intensity during activities on a Visual Analogue Scale (VAS), the RAND-36 Physical Component Scale (PCS) for general physical health, and the patient’s expectation for pain relief following treatment), and radiographic information (the Kellgren–Lawrence score for osteoarthritis [11], determined on a weight bearing radiograph in a posterior-anterior direction.

### Patient involvement

Ten patients were surveyed who were involved in the ESCAPE trial. The patients were asked to select relevant treatment selection factors according to their opinion. From the list of variables measured within the ESCAPE trial.

### Treatment outcome

The treatment outcome of interest was a clinically relevant improvement on patient reported knee function. For short-term effects, 3 months was identified by the patients and clinicians as an important time-point. Long-term effects were analysed at 12 and 24 months. Patient reported knee function was measured with the IKDC questionnaire [7]. The IKDC is a validated and reliable questionnaire with good responsiveness in patients with degenerative meniscal tears [17, 29]. Clinically relevant improvement was defined as an improvement exceeding the minimal important change (MIC) of the IKDC of 11 points for this patient population [17]. For this study, patients were divided into two groups at 3, 12, and 24 months follow-up: (1) patients who experienced a clinically relevant improved knee function (an improvement ≥ 11 IKDC points; i.e. good outcome) and (2) patients who did not experience a clinically relevant improved knee function (a deterioration or improvement < 11 IKDC points; i.e. poor outcome).

### Data processing and statistical analysis

For the preselected baseline characteristics with a continuous outcome, separate logistic regression models were developed (treatment selection models) to predict the outcome using (1) the baseline characteristic (marker), (2) the allocated treatment (meniscal surgery or physical therapy), and (3) a marker-by-treatment interaction term. Interactions with a *p* value for association < 0.1 were considered as potential treatment selection markers [3, 22].

These potential treatment selection markers were further explored using predictiveness curves. These predictiveness curves present the risk on a poor outcome (no clinically relevant improved or deteriorated knee function) for both treatments. Furthermore, they also provide information on the performance of the potential treatment selection markers to guide treatment decisions, so called summary measures [8]. A detailed explanation of a predictiveness curve is provided in Supplement 4. The performance of the potential treatment selection markers was analysed under the assumption that physical therapy is the standard treatment as suggested by the current guidelines [21, 24].

The summary measures provide information on:

1. *Marker positivity threshold*; the threshold value of the baseline score of the potential treatment selection marker. Above this value patients would receive a recommendation for physical therapy, below this value a recommendation for meniscal surgery;
2. *Marker positivity rate;* the proportion of patients with a marker value greater than the marker positivity threshold. For this proportion of the population, physical therapy has an advantage over meniscal surgery;
3. *Marker negativity rate*; the proportion of patients with a marker score smaller than the marker positivity threshold. For this proportion of the population, meniscal surgery has an advantage over physical therapy and for this group standard care (i.e. physical therapy) would be recommended to change;
4. *Average benefit physical therapy*; the average benefit of physical therapy in patients with a marker value above the marker positivity threshold. This measure evaluates the effect of physical therapy compared to meniscal surgery on the marker outcome;
5. *Average benefit meniscal surgery*; the average benefit of meniscal surgery in patients with a marker value below the marker threshold. This measure evaluates the effect of meniscal surgery, if treatment decision was guided by the model, in terms of the expected decrease in patients with a poor outcome;
6. *Decrease in rate of poor outcome*, the estimated change in the outcome in our population if treatment decisions are guided by the model compared to the outcome when treated according to standard care (i.e. physical therapy). This measure is used to provide information on the decrease in percentage of patients with a poor outcome when treatment is decided on basis of the treatment selection models.

All analyses were performed based on the intention-to-treat data. The data analyses for the predictiveness curves were performed using R-studio, version 1.2.1335 and package ‘Treatment selection’ (R-studio Inc., Boston, MA, USA) [8].

The sample size was determined and calculated for the RCT in which the patients’ data were collected. The details on the sample size calculations can be found in previous publications [26, 27].

## Results

### Participants

Three hundred and twenty-one patients were included in the study. The mean (SD) age was 57.5 (6.6) years, and 161 (50.5%) participants were female. A total of 158 patients were allocated to surgery and 161 to physical therapy. Both groups showed comparable baseline characteristics for the potential treatment selection markers (Table 1). Main results and a detailed flow chart of the ESCAPE trial were previously published [26].

**Table 1:** Patients’ baseline characteristics

	Surgery (*n* = 158)	Physical therapy (*n* = 161)
Demographics		
Age in years (SD)	57.6 (6.5)	57.3 (6.8)
Female (%)	80 (50.6)	81 (50.3)
Education level, high (%)	67 (42.4)	86 (53.4)
BMI (kg/m^2^) (SD)	26.7 (3.8)	27.2 (4.0)
Patient-reported outcomes		
Knee function on the IKDC (SD) 0–100, worse to best	44.8 (16.6)	46.5 (14.6)
General physical Health on the RAND-36 PCS (SD) 0–100, worse to best	37.6 (8.3)	37.9 (8.6)
Pain during activities on the VAS (SD) 0–100, best to worse	61.1 (24.5)	59.3 (22.6)
Expectation for pain relief (SD) 1–7, deterioration of pain to complete pain relief	5.6 (0.5)	5.3 (0.8)
Radiographic information^a^		
OA score on radiographs (K–L classification)^b^ (%)		
0—No OA	18 (12.0)	15 (10.1)
1—Doubtful	81 (54.0)	74 (49.7)
2—Minimal OA	45 (30.0)	55 (36.9)
3—Moderate OA	6 (4.0)	5 (3.3)
4—Severe OA^c^	0 (0%)	0 (0)
Tear location on MRI	*n* = 158	*n* = 161
Medial	126 (79.7)	136 (84.5)
Lateral	30 (19.0)	25 (15.5)
Both	2 (1.3)	0 (0)

Data are *n* (%) or mean [standard deviation (SD)]

Abbreviations: *BMI* body mass index, *IKDC* International Knee Documentation Committee Subjective Knee Subjective Knee, *PCS* Physical Component Score, *VAS* Visual Analogue Scale, *K–L* Kellgren–Lawrence classification, *OA* osteoarthritis, *MRI* Magnetic Resonance Imaging

^a^Surgery *n* = 150, Physical therapy *n* = 149

^b^Grade of knee osteoarthritis was assessed by X-ray using the Kellgren and Lawrence scale (K&L)

^c^K–L grade 4 was an exclusion criterion for participation in the ESCAPE trial

At 3 months follow-up, 57.0% (meniscal surgery) and 52.2% (physical therapy) of the patients were improved in knee function (> 11 IKDC points). At 12 months, this was 70.3% (surgery) and 54.7% (physical therapy), and at 24 months, this was 70.9% (surgery) and 65.8% (physical therapy). This shows that over time more patients achieved a clinically important improvement in knee function. In the physical therapy arm, 43 patients (27%) received delayed meniscectomy within 24 months.

### Treatment selection markers

Potential treatment selection markers at baseline were general physical health (*p* = 0.01), pain during activities (*p* = 0.02) and knee function (*p* = 0.07) for the outcome at 3 months; BMI (*p* = 0.05) and age (*p* = 0.06) for the outcome at 12 months; and age (*p* = 0.05) for the outcome at 24 months (Table 2).

**Table 2:** Logistic regression analyses for interaction between the baseline characteristics and treatment at 3, 12, and 24 months

Baseline characteristic	3 months (< MIC 139 vs. ≥ MIC 174)^a^		12 months (< MIC 80 vs. ≥ MIC 199)^a^		24 months (< MIC 71 vs. ≥ MIC 218)^a^	
	Marker-by-treatment interaction		Marker-by-treatment interaction		Marker-by-treatment interaction	
	OR^b^ (95% CI)	*p* value for interaction	OR^b^ (95% CI)	*p* value for interaction	OR^b^ (95% CI)	*p* value for interaction
Age	0.95 (0.89–1.02)	n.s. (0.14)	0.93 (0.84–1.00)	0.06*	0.92 (0.84–0.10)	0.05*
Education level (1–7)	1.02 (0.41–2.50)	n.s. (0.97)	0.65 (0.22–1.90)	n.s. (0.43)	1.01 (0.34–3.02)	n.s. (0.98)
BMI	0.93 (0.83–1.05)	n.s. (0.24)	0.86 (0.75–0.10)	0.05*	0.94 (0.82–1.09)	n.s. (0.41)
Knee function on the IKDC (0–100)	1.04 (0.10–1.07)	0.07*	1.01 (0.97–1.05)	n.s. (0.52)0	1.03 (0.99–1.08)	n.s. (0.16)
General physical health on RAND-36 PSC (0–100)	1.08 (1.02–1.15)	0.01*	1.05 (0.99–1.12)	n.s. (0.11)	1.04 (0.97–1.11)	n.s. (0.30)
Pain intensity during activities on VAS (0–100)	0.97 (0.95–0.10)	0.02*	0.99 (0.96–1.01)	n.s. (0.25)	0.10 (0.97–1.02)	n.s. (0.79)
Expectation of pain relief (1–7)	1.31 (0.63–2.71)	n.s. (0.47)	0.88 (0.38–2.06)	n.s. (0.77)	1.62 (0.65–4.07)	n.s. (0.30)
Knee osteoarthritis on K–L scale (0–4)^c^	0.71 (0.27–1.85)	n.s. (0.48)	0.99 (0.32–3.11)	n.s. (0.99)	1.22 (0.39–3.87)	n.s. (0.74)

Abbreviations: *MIC* minimally important change, *OR* odds ratio, *CI* confidence intervals, *IKDC* International Knee Documentation Committee Subjective Knee, *RAND-36 PCS* Physical Component Scale for or general physical health, *BMI* body mass index, *K–L* Kellgren–Lawrence scale

Marker-by-treatment interactions per follow-up moment are shown

^a^(*n* =  < MIC vs. *n* =  ≥ MIC) For each follow-up moment the distribution of patients who experienced MIC in knee function (improvement ≥ 11 IKDC points) and patients who did not experience a MIC in knee function (changed IKDC score < 11 points) is reported. The reference treatment is physical therapy. Data were available of 313 patients at 3 months, 279 patients at 12 months, and 289 patients at 24 months

^b^For each marker-by-treatment interaction, the OR shows the relative change per unit increase in the marker and we reported the 95% CI of the OR. An OR ≥ 1 indicates the value is in favour of physical therapy. The *p* values expressed whether the marker-by-treatment interaction is significant (*p* ≤ 0.1)

^c^We analysed educational level, expectation of pain relief an K–L score as a continuous variable in the logistic regression analyses

*Indicates the baseline characteristics that are potential treatment selection markers

### Prediction curves for potential treatment selection markers

**Fig. 1:**
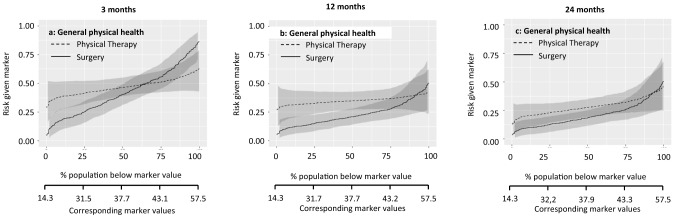
Patients with a score above the threshold would improve more from physical therapy. The marker-by-treatment interaction at 3 months is significant (*p* = 0.01) with a corresponding marker positivity threshold of 40.7 points. At 12 and 24 months follow-up the marker-by-treatment interactions are no longer significant. Therefore, general physical health is not useful for treatment selection on the longer term.

**Fig. 2:**
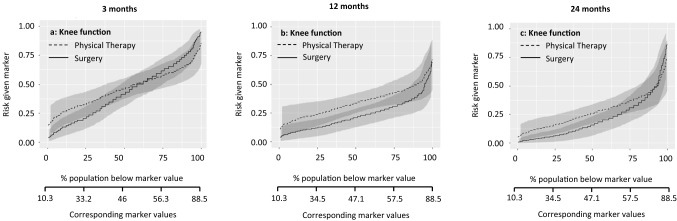
Patients with a score above the threshold would improve more from physical therapy. The marker-by-treatment interaction at 3 months is significant (*p* = 0.07) with a corresponding marker positivity threshold of 50.6 points. At 12 and 24 months follow-up the marker-by-treatment interaction are no longer significant. Therefore, knee function is not useful for treatment selection on the longer term

**Fig. 3:**
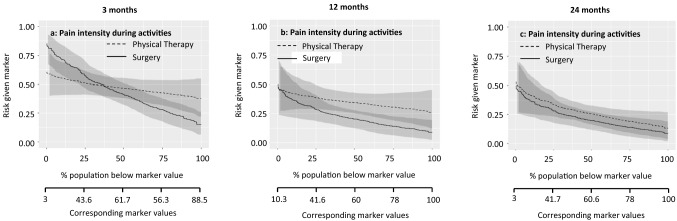
Patients with a score above the threshold would improve more from physical therapy. The marker-by-treatment interaction at 3 months is significant (*p* = 0.07) with a corresponding marker positivity threshold of 53.9 points. At 12 and 24 months follow-up the marker-by-treatment interaction are no longer significant. Therefore, pain intensity during activities is not useful for treatment selection on the longer term

**Fig. 4:**
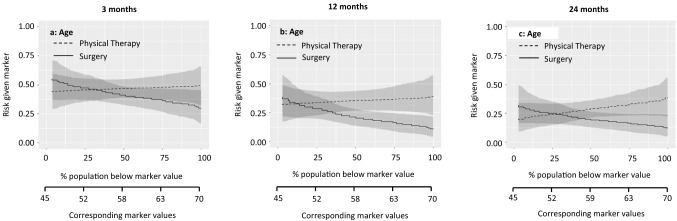
Patients with a score above the threshold would improve more from meniscal surgery. The marker-by-treatment interaction for the marker age is not significant at 3 months. However, at 12 and 24 months follow-up the marker-by-treatment interaction are significant (12 months *p* = 0.06; 24 months *p* = 0.05). The corresponding marker positivity threshold at 12 months follow-up is 49 years old and at 24 months follow-up the marker positivity threshold is 53 years old

**Fig. 5:**
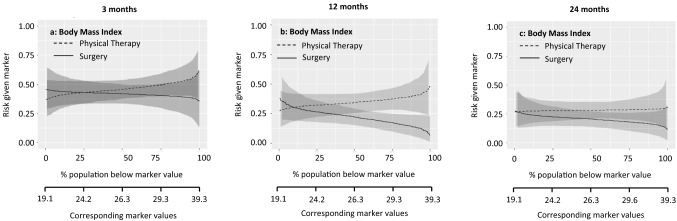
Patients with a score above the threshold would improve more from meniscal surgery. The marker-by-treatment interaction for body mass index is not significant at 3 months. At 12 follow-up the marker-by treatment interaction is significant (*p* = 0.05) with corresponding marker positivity threshold of 22.3. However, at 24 months follow-up the marker-by-treatment interaction are no longer significant. Therefore, body mass index is not useful for treatment selection on the short and long term

These potential treatment selection markers were further explored with predictiveness curves. Figures 1, 2, 3, 4, 5 show the predictiveness curves at 3, 12, and 24 months for the following markers: general physical health, knee function, pain intensity during activities, age, and BMI.

Marker-by-treatment predictiveness curves for the outcome at 3, 12, and 24 months. Abbreviations: *PT* physical therapy, *APM* arthroscopic partial meniscectomy (surgery); predictiveness curves present the risk for individual patients, with a certain marker score, at the outcome of interest due to the given treatment. On the X-axis is the proportion of patients displayed that score below the corresponding marker value. The corresponding marker value is the raw marker score. On the Y-axis is the risk for the individual patient at the outcome with physical therapy and arthroscopic partial meniscectomy displayed.

The sloping lines in Fig. 1 indicate that the risk of poor outcome increases with higher levels of baseline general physical health. This applies to both treatments. The intersection in Fig. 1a indicates that at 3 months, patients with baseline values below 40.7/100 were more likely to benefit from surgery and patients with baseline values above 40.7/100 were more likely to benefit from physical therapy. The curves at 12 and 24 months follow-up were sloping less, indicating a similar risk of poor outcome across baseline values. Figure 2 shows a similar pattern for the marker knee function. The curves at 12 and 24 months run largely parallel, indicating that the effect of this baseline marker was similar for both treatments. The lines sloping down in Fig. 3 indicate that the risk of poor outcome decreased with higher levels of pain at baseline. The intersection in Fig. 3a indicates that at 3 months, patients with baseline VAS scores below 53.9/100 were more likely to benefit from surgery and patients with baseline VAS scores above 53.9/100 were more likely to benefit from surgery. No such intersection indicating a potentially relevant cut-off for baseline pain was observed at 12 and 24 months. For age and BMI, all curves were rather horizontal, but diverging with increasing baseline marker values with half of them reaching statistical significance. This indicates that the benefit of surgery compared to physical therapy was largest for patients with highest age and BMI. For all markers the predictiveness curves were inconsistent over time.

The summary measures of the predictiveness curves are presented in Table 3. This provides information on marker positivity threshold, average benefit physical therapy, average benefit surgery, marker positivity rate, marker negativity rate, and decrease in rate of poor outcome.

**Table 3:** Summary measures of predictiveness curves for pain during activities

	3 months	12 months	24 months
**General physical health**			
Marker positivity rate	36.8% (5.4–66.1)	8.9% (0.0–36.5)	7.3% (0.0–48.5)
Marker negativity rate	63.2% (33.5–94.6)	91.1% (62.7–100)	92.7% (51.2–100
Marker positivity threshold	40.7	51.0	52.0
Average benefit physical therapy	10.1% (1.1–20.6)	0.3% (0.0–15.7)	1.8% (0.0–14.1)
Average benefit surgery	13.1% (4.1–22.9)	13.8% (5.2–24.1)	8.4% (1.8–17.5)
Decrease in rate of poor outcome	8.3% (1.5–16.4)	12.6% (3.8–22.5)	7.7% (1.3–16.7)
**Knee function**			
Marker positivity rate	37.9% (− 0.1 to 85.5)	0% (0.0–38.4)	8.7 (0.1–54.4)
Marker negativity rate	62.1% (14.2–100)	100% (60.2–100)	91.3% (44.6–99.9)
Marker positivity threshold	50.6	NA	65.5
Average benefit physical therapy	6.0% (0.0–15.0)	0.1% (0.1–10.8)	5.8% (0.1–17.0)
Average benefit surgery	8.9% (1.0–18.6)	11.3% (3.1–21.9)	8.2% (1.4–17.6)
Decrease in rate of poor outcome	5.5% (0.1–14.5)	11.3% (2.0–21.6)	7.4% (1.0–16.5)
**Pain intensity during activities**			
Marker positivity rate	62.8% (30.7–97.6)	99.2% (63–100)	99.9% (0.1–99.9)
Marker negativity rate	37.2% (2.0–68.6)	0.8% (0.0–36.6)	0.1% (0.1–100)
Marker positivity threshold	53.9	9.3	NA
Average benefit physical therapy	11.0% (0.3–22.4)	1.4% (0.0–14.0)	0% (0.0–12.2)
Average benefit surgery	12.2% (2.7–23.7)	12.7% (3.8–23.9)	5.2% (0.0–16.7)
Decrease in rate of poor outcome	7.7% (0.9–16.8)	12.6% (3.1–22.7)	5.2% (0.0–15.6)
**Age**			
Marker positivity rate	67.4% (19.8–100)	88.8% (60.5–99.9)	72.2% (48.7–99.9)
Marker negativity rate	32.6% (0.0–79.3)	11.2% (0.1– 39.5)	27.8% (0.1–50.9)
Marker positivity threshold	54.0	49.0	53.0
Average benefit physical therapy	5.0% (0.0–16.7)	4.1% (0.0–14.4)	5.5% (0.0–17.0)
Average benefit Surgery	9.5% (1.2–20.4)	14.8% (6.5–25.7)	12.8% (4.3–22.0)
Decrease in rate of poor outcome	6.4% (0.3–16.2)	13.2% (0.5–23.7)	9.3% (2.8–18.2)
**Body mass index**			
Marker positivity rate	75.7% (6.3–99.9)	90.3% (52.7–100)	99.0% (30.8–100)
Marker negativity rate	24.3% (0.1–93.7)	9.7% (0.0–46.9)	1.0% (0.0–68.9)
Marker positivity threshold	24.2	22.3	19.6
Average benefit physical therapy	3.2% (0.0–13.6)	4.4% (0.0–12.7)	0.5% (0.0–8.7)
Average benefit surgery	7.3% (0.1–19.2)	14.8% (6.7–25.1)	8.0% (1.3–18.3)
Decrease in rate of poor outcome	5.5% (0.1–16.1)	13.4% (5.4–23.6)	7.9 (0.4–17.9)

The proportions are given in percentages (95% confidence interval)

Abbreviation: *NA* not available, no marker positivity threshold as the line do not cross each other)

The score ranges from 0 to 100, with 100 representing the best possible knee function

Interpretation (example): For general physical health at 3 months, 36.8% (95% CI 5.4–66.1) of the patients scored higher than the threshold value of 40.7 points (marker positivity rate), representing the cut-off point for a better outcome from physical therapy. Patients with a score above this threshold had an average 10.1% (95% CI 1.1–20.6) better outcome from physical therapy as compared to those treated with surgery (average benefit physical therapy). A total of 63.2% (95% CI 33.5–94.6) of the patients scored lower than the threshold (marker negatively rate). These patients had an average 13.1% (95% CI 4.1–22.9) better outcome from surgery as compared to those treated with physical therapy (average benefit surgery). If treatment would be based on general health, there would be an 8.3% (95% CI 1.5–16.4) reduction in poor outcomes at 3 months if all patients with a RAND-36 score below 40.7 would receive surgery (decrease in rate of poor outcome)

## Discussion

The important finding of the present study was that the identification of potential treatment selection markers did not result in clear clinical subgroups of patients who are substantially more likely to benefit from either surgery or physical therapy. Therefore, treatment decisions for patients with degenerative meniscal tear cannot be based on these treatment selection markers evaluated in the current study.

The published randomized clinical trials that evaluated surgical to conservative treatment for degenerative meniscal tears revealed small and clinically non-meaningful benefits of meniscal surgery over physical therapy in patients with degenerative meniscal tears for patient reported knee function [5, 6, 9, 15, 26, 32]. However, due to potential heterogeneity in treatment effects, this does not necessarily imply that individual patients cannot have a clinically relevant improvement from meniscal surgery compared to physical therapy [23].

The present study revealed that the average benefit that individual patients would experience from meniscal surgery is small (ranging from 5.2 to 14.8%) if treatment would be based on these markers. Similar to the results from the RCTs, the increased benefit that some patients may experience from meniscal surgery compared to physical therapy is not convincing since these benefits were small and not consistently present on all follow-up moments.

No studies were found that have analysed the variation in treatment effect for a musculoskeletal disorder based on RCT baseline data of their patients by performing a marker-by-treatment analysis. One cohort study on prognostic factors was identified and found worse outcomes at 1 and 2 years after surgery in case of complex tears, larger extrusion, cartilage injuries, and larger meniscal excision but without comparison to physical therapy [14]. In another, computer-based, prediction model in a similar population multivariable prognostic models were investigated to identify a subgroup of patients who might benefit from meniscectomy [19]. The multivariable prognostic models did not accurately predict treatment outcome after 1 year of surgery, and the study did not consider specific cut-off points that can potentially differentiate between the outcomes from the two treatments. In another study the orthopaedic surgeons’ prediction ability for treatment outcome in patients with degenerative meniscal tears was analysed for both physical therapy and meniscectomy [25]. Similar to the current findings, neither of these prediction studies were able to identify any subgroup of patients who might benefit from a meniscectomy or physical therapy on the longer term [25].

To our knowledge, this is the first RCT-based marker-by-treatment analysis that assessed the differential treatment effect of potentially relevant baseline variables for predicting clinically relevant improvement of knee function in patients with a degenerative meniscal tear. For treatment decision making, this type of prediction studies may be favourable over more common multivariable prediction studies [8]. Marker-by-treatment analyses focus on predicting the difference in outcomes between the two treatments, rather than only predicting the outcome for one treatment. Therefore, the analyses help clinicians and patients select the best treatment to optimize the outcomes. The selection markers deemed relevant by patients in this study aim to direct the choice of treatment based on specific baseline characteristics and the corresponding marker cut-off values.

Several limitations should be mentioned. First, the observed treatment thresholds did not account for potential adverse events resulting from surgery as an alternative to physical therapy. In other words, treatment benefit from surgery is overestimated because the risk of surgical complications is neglected [2]. Second, due to trial-based approach, the available baseline characteristics were restricted. Some potentially important predictors, such as objective knee function, muscle strength, the duration of symptoms [10], could not be included in our analyses. Although these factors may be viewed as relevant for treatment outcome, prior prognostic models that did include these variables could also not accurately predict treatment outcome in this population [19]. [10] Trials that included these prognostic variables are recommended to perform a marker-by-treatment analysis including these variables. Also, the trial-based approach might have resulted in an insufficient power for marker-by-treatment analysis due to the size of the RCT cohort. Third, the primary interest concerned the cut-off point on a predictiveness curve that distinguishes between a better outcome after surgery or after physical therapy based on a patient’s baseline score. Therefore, only continuous variables could be included, and dichotomous and categorical variables such as sex, joint line tenderness and tear type were not addressed.

### Clinical implications

In general, marker-by-treatment analyses determine whether baseline characteristics can be used in making treatment decisions for individual patients. The predictiveness curves and performance measures of these predictiveness curves show the amount of benefit that individual patients will have from a treatment, if the treatment decision for that patient is based upon the treatment selection marker [8]. A threshold value is derived that differentiates between a favourable outcome for either of the compared treatments. Although such thresholds are rather uncommon to use as treatment decision tool in clinical practice, this information can be of high value to clinicians and policy makers who are seeking evidence based decision tools to weigh treatment benefit for individual patients against the risk of adverse events and healthcare costs [13]. So, instead of using mean outcomes of RCTs to make treatment decisions, patients and clinicians can potentially base their treatment decision for an individual patient upon the treatment selection marker.

In patients with degenerative meniscal tears our marker-by-treatment analyses only revealed specific baseline characteristics that showed a small increase in a better treatment outcome after meniscal surgery for each follow-up time point. As the combination of characteristics varies among patients, combining these potential selection markers may be more accurate. Future research, combining the individual data from the published RCTs in an individual patient data meta-analysis [31], may be able to identify any of these subgroups and could steer towards an even more individualized approach.

The opinion that surgery is necessary to regain normal knee function in selected patients is not supported by our study or previous scientific evidence in which subgroups have been unable to be identified [4, 19, 25]. Therefore physical therapy is recommended as initial treatment for all patients with degenerative meniscal tears.

## Conclusion

A marker-by-treatment analysis was successfully conducted in orthopaedic research. No subgroups were found in this study that benefit more from surgery throughout the follow-up period. Physical therapy should be considered first choice treatment in all patients over 45 years old with degenerative meniscal tears who do not experience locking of the knee. Although the treatment selection markers had clear thresholds, none of the markers maintained a predictive effect over time. Therefore, treatment decisions for patients with degenerative meniscal tear cannot be based on the treatment selection markers studied in this trial.

## Supplementary Information

Below is the link to the electronic supplementary material.

Supplementary file1 (DOCX 95 KB)

## Data Availability

Requests for access to data should be addressed to the corresponding author.

## Declarations

### Transparency

The corresponding author affirms that the manuscript is an honest, accurate, and transparent account of the study bring reported; that no important aspects of the study have been omitted; and that any discrepancies from the study as planned (and, if relevant, registered) have been explained.
