# Supplementary material for: An individualized decision between physical therapy or surgery for patients with degenerative meniscal tears cannot be based on continuous treatment selection markers: a marker-by-treatment analysis of the ESCAPE study
Source: Knee Surg Sports Traumatol Arthrosc. 2022 Feb 5;30(6):1937–48. doi: 10.1007/s00167-021-06851-x (PMC9165275; doi:10.1007/s00167-021-06851-x)
Supplement: Supplementary file 1 — Supplementary file1 (DOCX 95 KB) [file 167_2021_6851_MOESM1_ESM.docx]

**Supplement 1. Physical therapy exercise program.**

__________________________________________________________________________________

Time Exercises Repetitions or time

(week)

__________________________________________________________________________________

**0-8** Stationary cycling for warming up 15 min or longer and cooling down or cardiovasculair training *gradually increase intensity*

**0-8** Pully or dynaband, strapped around ankle uninjured side. 3x12 reps

Stand on injured side and keep balance, *each direction*

Step with uninjured side forward, backwards and sideways

**0-4** Calf raises on a leg press machine 3x12 reps

**0-8** Hamstrings: standing hip extension in a 3x12 reps
 “multi-hip” trainings device

**0-4** Keeping balance on a balance board, use both feet 30 – 60 sec.

**0-8** Climbing stair, walking, acceleration, running, 10 min

Jumping. *According to the patient’s activity level*

**5-8** Calf raises standing on one leg 3x12 reps

**1-8** Leg press, place feet high enough for the 3x12 reps
shinbones to become in a horizontal plane
and the knee starting at 110˚ flexion, unilateral

**5-8** Squats (according to the needs of the patient) 3x12 reps

In which a knee flexion > 90° is not allowed

**5-8** Balance board on one foot 3 min

challenge with throwing a ball

**5-8** Elliptical machine for warming up 10 min or longer and cooling down or cardiovascular training

**The exercise program contained 16 supervised sessions during 8 weeks**

For all exercises is it important to keep the patients’ individual needs and limitations focused by using the ICF. The uninjured side is also less trained as usual and therefore both sides should be trained.
Besides training of the lower extremity, “core stability” training is of importance for good posture positioning and moving. The active rehabilitation program is designed around cardiovascular (circulation), coordination and balance, and closed chain strength exercises. Shearing forces in the knee are less using closed chain exercises compared to open chained exercises. The closed chain exercises activate both agonists and antagonists around the knee joint resulting in a direct rotatory movement and prevent in shearing forces seen by open chained exercises.

**Home exercise program**
In addition, a home exercise program was provided to all participants. It consisted of one leg standing during 60 seconds and a step-down exercise comprising 3, 9, 10 repetitions, twice a week.

**Supplement 2. List of variables measured in the ESCAPE trial**

**Patients’ demographics**

- Age*
- Sex
- BMI*
- education level*
- Smoking status
- Employment

**Physical examination**

- McMurray test
- Range of motion
- Joint effusion
- Duck walk test
- Thessaly test
- Pain at full flexion
- Pain at full extension
- Jointline tenderness
- Circumference Femur

**Radiographic information (X-ray and MRI**

- Grade of osteoarthritis of the knee*
- Rupture of Anterior cruciate ligament in medical history
- Presence of bakers’ cyct

**Patient reported outcome measures**

- Patient specific functioning scale
- RAND-36 for general health*
- Tegner activity scale
- International Knee Documentation Committee Subjective Knee Form (IKDC) for knee function*
- Euroqol 5 dimension, 5 level (EQ5D5L)
- Pain intensity in rest on a Visual Analogue Scale (VAS)
- Pain intensity during activities on VAS*
- Patients’ expectation for pain relief following treatment*

*Variables marked with an asterisk were selected as potential treatment selection markers.*

**Supplement 3 *Search strategy***

Search (((("Meniscus"[Mesh] OR menisc*[tiab]) AND (tear*[tiab] OR injur*[tiab] OR lesion*[tiab] OR rupture*[tiab])) AND ("Conservative Treatment"[Mesh] OR "Rehabilitation"[Mesh] OR "Physical Therapy Modalities"[Mesh] OR "Physical Therapy Department, Hospital"[Mesh] OR "Exercise"[Mesh] OR "Exercise Movement Techniques"[Mesh] OR "Exercise Therapy"[Mesh] OR "Physical Therapy (Specialty)"[MeSH] OR rehabilitati*[tiab] OR physiotherap*[tiab] OR ((physical[tiab] OR conservative[tiab]) AND (therapy[tiab] OR therapies[tiab] OR activity[tiab] OR activities[tiab] OR treatment*[tiab] OR management*[tiab])) OR exercis*[tiab] OR training*[tiab]) AND ("Epidemiologic Studies"[Mesh] OR cohort[tiab] OR (case[tiab] AND (control[tiab] OR controll*[tiab] OR comparison[tiab] OR referent[tiab])) OR risk[tiab] OR causation[tiab] OR causal[tiab] OR "odds ratio"[tiab] OR etiol*[tiab] OR aetiol*[tiab] OR "natural history"[tiab] OR predict*[tiab] OR prognos*[tiab] OR outcome[tiab] OR course[tiab] OR retrospect*[tiab]))) AND ((("Patient Satisfaction"[Mesh] OR patient satisfaction[tiab] OR satisfaction[tiab])) OR (cross over) OR (IKDC[tiab] OR outcome*[tiab] OR KOOS[tiab] OR WOMAC[tiab]))

**Supplement 4. An example of a predictiveness curve with summary measures**


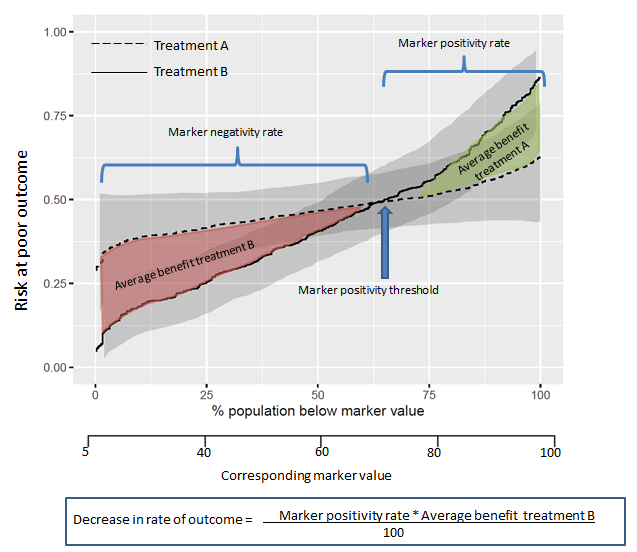


Predictiveness curves present the risk for individual patients, with a corresponding marker value, at the outcome of interest due to the given treatment. This example uses fictitious numbers and provides a manual to interpret the study results that are presented in the manuscript.

In this example treatment A is the reference treatment. The outcome is a dichotomous outcome, corresponding to a poor outcome. The graph displays the risk at a poor outcome.

The **X-axis,** the proportion of patients with a value or score below the corresponding marker value. The **corresponding marker value** is the value of that marker scored by an individual patient.

The **Y-axis,** represents the risk for the individual patient at a poor outcome when undergoing treatment A or treatment B.

**Marker positivity threshold**, the intersection of both treatments, in this example the corresponding marker value is 75;

**Marker positivity rate,** the proportion of patients with a marker score greater than the marker positivity threshold, in this example this is the case in 40% of the patients. For this proportion of patients treatment A has an advantage over treatment B;

**Marker negativity rate,** the proportion of patients with a marker score lower than the marker positivity threshold, in this example this is the case in 60% of the patients. These patients have a higher risk at a poor outcome due to the standard treatment A. For this proportion of the population, treatment recommendation would change;

The **average benefit of treatment A**. In this example the average benefit of treatment A is 10%. This means that we would expect an average decrease of 10% of patients with a poor outcome due to treatment A.

The **average benefit of treatment B**. In this example the average benefit of treatment B is 15%. This indicates that we would expect an average decrease of 15% of patients with a poor outcome due to treatment B.

The **decrease in rate of outcome** is the reduction in poor outcome when patients avoid treatment A and follow treatment recommended by the model. This is calculated by multiplying the marker negativity rate of 60% with the average benefit of treatment B for this subgroup of 15%, divided by 100. In this example, this has the value of 60%*15% / 100 = 9%. This means an almost 9% reduction in poor outcomes when all patients with a baseline score exceeding the marker positivity threshold are given treatment B.
